# Antiarrhythmic Effect of Ranolazine in Combination with Selective NCX-Inhibition in an Experimental Model of Atrial Fibrillation

**DOI:** 10.3390/ph13100321

**Published:** 2020-10-20

**Authors:** Julian Wolfes, Christian Ellermann, Niklas Broer, Benjamin Rath, Kevin Willy, Patrick Robert Leitz, Philipp Sebastian Lange, Lars Eckardt, Gerrit Frommeyer

**Affiliations:** Department of Cardiology II (Electrophysiology), University Hospital Münster, Albert-Schweitzer-Campus 1, 48149 Münster, Germany; christian.ellermann@ukmuenster.de (C.E.); ni_bro@live.com (N.B.); benjamin.rath@ukmuenster.de (B.R.); kevin.willy@ukmuenster.de (K.W.); patrickrobert.leitz@ukmuenster.de (P.R.L.); Philippsebastian.lange@ukmuenster.de (P.S.L.); lars.eckardt@ukmuenster.de (L.E.); gerrit.frommeyer@ukmuenster.de (G.F.)

**Keywords:** ranolazine, NCX-inhibition, atrial fibrillation, Langendorff

## Abstract

The aim of this study was to investigate the effects of a combination of ranolazine with different selective inhibitors of the Na^+^/Ca^2+^-exchanger (NCX) in an established experimental model of atrial fibrillation (AF). Eighteen hearts of New Zealand white rabbits were retrogradely perfused. Atrial catheters were used to record monophasic action potentials (aPRR). Hearts were paced at three different cycle lengths. Thereby, atrial action potential durations (aAPD_90_), atrial effective refractory periods (aERP) and atrial post-repolarization refractoriness were obtained. Isoproterenol and acetylcholine were employed to increase the occurrence of AF. Thereafter, the hearts were assigned to two groups *(n* = 9 each group) and additionally perfused with a combination of 10 µM ranolazine and 1 µM of the selective NCX-inhibitor ORM-10103 (group A: Rano-ORM) or 10 µM ranolazine and 1 µM of another NCX-inhibitor, SEA0400 (group B: Rano-SEA). The infusion of Iso/ACh led to a shortening of aAPD_90_, aERP, aPRR and the occurrence of AF episodes was significantly increased. Additional perfusion with ranolazine and ORM-10103 (group A) significantly prolonged the refractory periods and aPRR and AF episodes were effectively reduced. In group B, Rano-SEA led to a slight decrease in aAPD_90_ while aERP and aPRR were prolonged. The occurrence of AF episodes was consecutively reduced. To our knowledge, this is the first study investigating the effect of ranolazine combined with different selective NCX-inhibitors in an isolated whole-heart model of AF. Both combinations prolonged aERP and aPRR and thereby suppressed the induction of AF.

## 1. Introduction

Treatment of atrial fibrillation (AF) remains challenging and the results of antiarrhythmic drugs (AADs) in atrial fibrillation are often unsatisfactory for both patients and doctors [1]. The few AADs available are often limited to narrow indications. Furthermore, most AADs come with severe side effects including the risk of proarrhythmia. Although AF is the most common sustained arrhythmia, the development of new antiarrhythmic drugs proceeds only slowly and the common AADs are often in the market for multiple decades [2].

Consequently, the development of new AADs or the use of drugs, approved for different indications, have great clinical relevance. Ranolazine, an FDA-approved antianginal agent which influences cardiac electrophysiology by inhibiting I_Na_, I_NaL_, I_Kr_ [3] and TASK-1 [4] with a certain degree of atrial selectivity and a low risk of proarrhythmia [5], is a potential new compound to treat AF. While proving its antiarrhythmic effects in the treatment of AF after cardiac surgery [6], ranolazine has failed to terminate persisting forms of AF [7]. To improve the therapeutic effectivity of ranolazine in AF treatment, drug-combinations with other AADs [8] or novel compounds [9] are currently under investigation. A new target in AF is the Na^+^/Ca^2+^ exchanger (NCX), which is crucial in generating triggered activity from Ca^2+^ release events [10], while it was shown that NCX is upregulated in different models of AF [11,12]. NCX can be targeted with different molecules. SEA-0400 inhibits NCX while having a significant inhibitory effect on I_CaL_ [13], in contrast ORM-1010103 shows higher selectivity for cardiac NCX [14].

This study aimed to investigate the potential synergistic effects of combining ranolazine with different NCX-inhibitors in an established experimental model of AF.

## 2. Results

**Figure 1:**
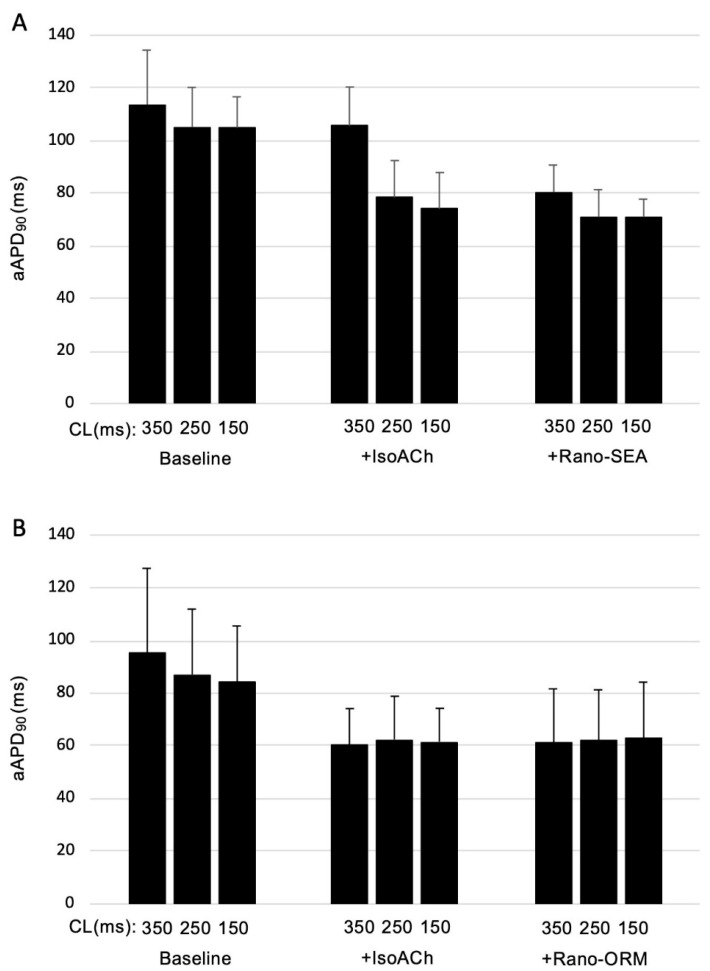
CL-dependent effects on atrial action potential duration (aAPD_90_) under baseline conditions and after the infusion of isoproterenol acetylcholine and the additional infusion of ranolazine/SEA0400 (*n* = 9) (**A**) or ranolazine/ORM-10103 (*n* = 9) (**B**).

Perfusion with isoproterenol and acetylcholine changed electrophysiologic parameters in both groups. The aAPD, aERP, and aPRR were shortened. In the Rano-ORM group, aAPD was shortened from 88.8 ± 26.2 ms to 61.5 ± 14.3 ms (*p* < 0.05) and in the Rano-SEA group from 107.9 ± 15.8 ms to 85.9 ± 14.2 ms (*p* < 0.05). Figure 1 illustrates the effects of IsoACh and Rano-ORM and Rano-SEA on aAPD_90_.

**Figure 2:**
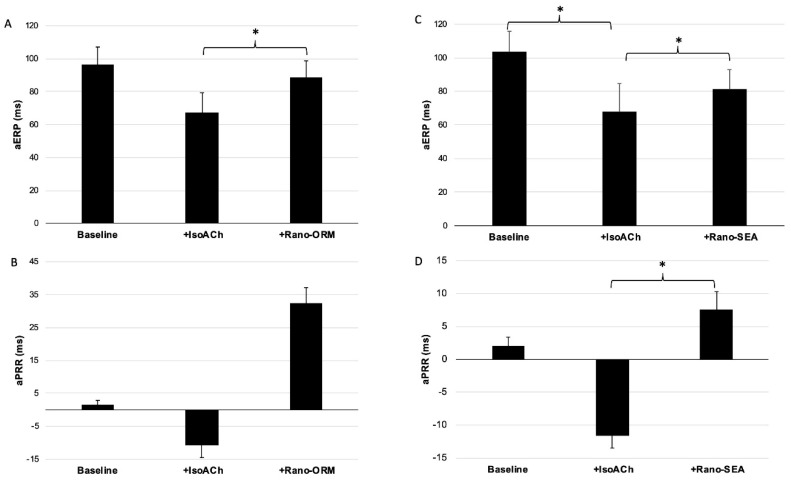
(**A**,**B**) the influence of additional perfusion with isoproterenol/acetylcholine and ranolazine/ORM-10103 on aERP (*n* = 9) (**A**) and atrial post repolarization refractoriness (aPRR) (*n* = 9) (**B**) (*: *p* < 0.05). (**C**,**D**) the influence of additional perfusion with isoproterenol/acetylcholine and ranolazine/SEA-0400 on aERP (*n* = 9) (**C**) and aPRR (*n* = 9) (**D**) (*: *p* < 0.05).

In group B, the treatment led to a significant reduction of the aERP (103.3 ± 12.4 ms to 67.8 ± 16.8 ms (*p* < 0.05)) whereas in group A the trend was similar, but the results were not significant (96.7±10.4 ms to 67.5 ± 11.8 ms (*p* = ns)). Consequently, the aPRR trend to shortened values went from 1.5 ± 1.4 ms to −10.7 ± 3.8 ms (*p* = ns) in group A and from 1.9 ± 1.4 ms to −11.7 ± 1.8 ms (*p* = ns) in group B, without reaching the threshold for statistical significance. The influence of the different perfusates on aERP and aPRR is summarized in Figure 2.

**Figure 3:**
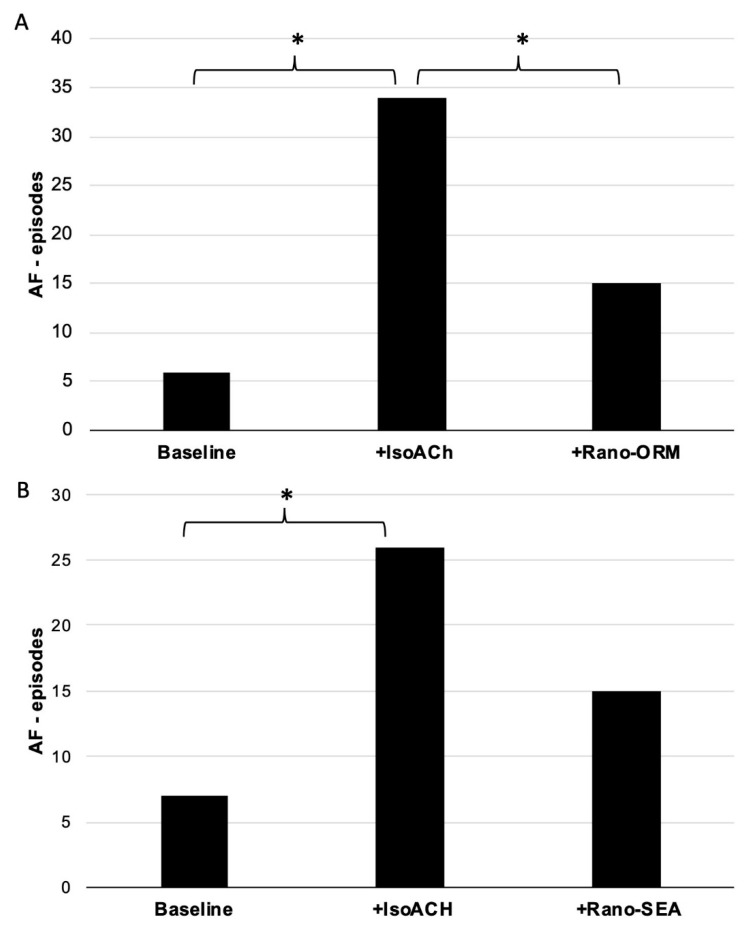
(**A**) total number of induced episodes of atrial fibrillation (AF) under baseline conditions and after perfusion with isoproterenol/acetylcholine and with ranolazine/ORM-10103 (*n* = 9) (*: *p* < 0.05). (**B**) total number of induced episodes of atrial fibrillation under baseline conditions and after perfusion with isoproterenol/acetylcholine and with ranolazine/SEA0400 (*n* = 9) (*: *p* < 0.05).

**Figure 4:**
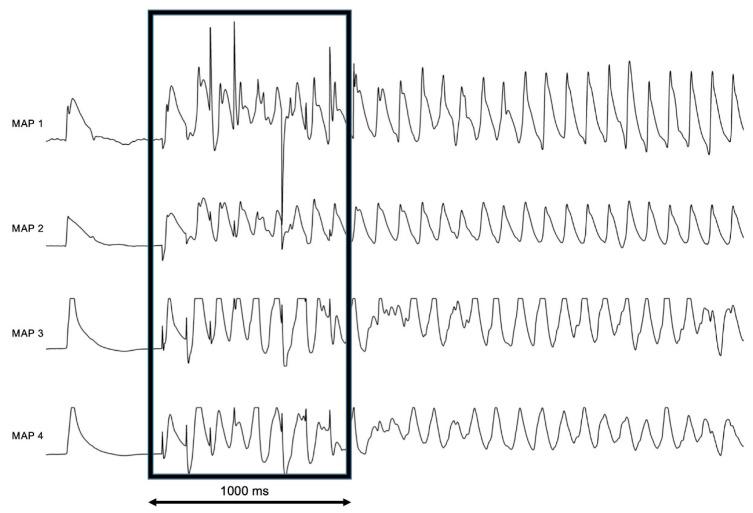
Representative example of the monophasic action potentials (MAP)-traces (MAP 1–4) of atrial fibrillation induced by burst pacing (box) under perfusion with isoproterenol and acetylcholine, 1000 ms for scale.

Perfusion with IsoACh led to a significant increase in the number of inducible episodes of AF (group A: six episodes at baseline to 34 episodes *p* < 0.05; group B: seven episodes at baseline to 26 episodes under IsoACh (*p* < 0.05)) as shown in Figure 3. An example of AF induced by programmed stimulation is shown in Figure 4.

After the application of both combinations (Rano-ORM and Rano-SEA), the mean aAPD was unchanged in group A (61.5 ± 14.3 ms to 61.8 ± 20.6 ms, *p* < 0.05). In group B, additional perfusion with Rano-SEA led to a further slight decrease in aAPD_90_ (85.9 ± 14.2 ms to 74.0 ± 9.2 ms, *p* < 0.05).

In both groups the combination of ranolazine with selective NCX-inhibitors induced a significant prolongation of aERP and aPRR. The additional perfusion with Rano-ORM prolonged aERP from 67.5 ± 11.8 to 88.9 ± 9.8 ms (*p* < 0.05), consequently aPRR was increased from −10.7 ± 3.8 ms to 32.4 ± 4.8 ms (*p* < 0.05). In group B, additional perfusion with Rano-SEA prolonged aERP from 67.8 ± 16.8 ms to 81.1 ± 11.8 ms (*p* < 0.05). As a result, aPRR was prolonged from −11.7 ± 1.8 ms to 7.5 ± 2.8 ms (*p* < 0.05).

The inducibility of AF was reduced in both groups by the additional perfusion with ranolazine and the selective NCX-inhibitors. In group A, Rano-ORM significantly reduced the number of AF episodes from 34 under IsoACh to 15 (*p* < 0.05). Rano-SEA also reduced the inducibility of AF from 26 to 15 (*p* = 0.1).

## 3. Discussion

The results of the present study demonstrate for the first time the potential antiarrhythmic effect of ranolazine in combination with different NCX-inhibitors in an intact whole-heart model of AF. An increase in the atrial effective refractory period with a consecutively increased aPRR in the setting of a mostly unaltered aAPD seems to be crucial for the antiarrhythmic effect of ranolazine in combination with NCX-inhibitors in the given model. As shown previously, a prolonged PRR can shield the atria against premature excitation and re-entry [8,15]. Noteworthy is that the narrowing of the aAPD is often observed in persisting AF due to electrical remodelling, thereby aPRR prolongation seems a worthwhile mechanism in AF therapy.

To our best knowledge, this is the first study evaluating a combination of ranolazine and selective NCX-inhibitors in a rabbit whole-heart model of AF.

### 3.1. Drug Combination in Antiarrhythmic Therapy

Ranolazine is an FDA-approved antianginal agent with already known antiarrhythmic effects on the ventricular and atrial level in this [16,17] model, experimental models of other groups [9] and clinical trials [18].

Ranolazine’s effect on the molecular electrophysiology has been investigated in previous studies. Ranolazine acts as a multi-ion-channel blocker, blocking late and peak I_Na_, I_Kr_ [3], and as recently shown, the nearly atrial specific two-pore domain potassium channel TASK-1 [4]. Sossalla et al. [19] demonstrated altered sodium channel kinetics with increased late I_Na_ (I_NaL_) in atrial appendages from patients with AF and furthermore showed the potency of ranolazine to suppress the altered late sodium influx. Inhibitory effects on sodium influx, especially peak I_Na_, might be founding for the prolonged aERP and aPRR observed in our experiments. Noteworthy, sole I_NaL_ inhibition usually shortens ERP without preventing AF [20].

In contrast to other antiarrhythmic drugs, ranolazine bears a low risk of proarrhythmia and has rather mild side-effects [21]. Despite these positive effects, sole ranolazine infusion has been shown to be ineffective in terminating persisting forms of AF [7]. In this light, combining ranolazine with other AADs has been employed in different models [8,9]. While ranolazine was combined with dronedarone in a large clinical trial [22], the study presented focused on the combination of ranolazine with the rather novel target of NCX-inhibition in AF.

Previous studies have shown diastolic calcium concentration partially resulting from raised SR-calcium release [23] leading to inward I_NCX_ and I_NCX_-upregulation [24] often resulting in delayed after depolarization-triggering AF. The combined AF-induction applied in this study, consisting of perfusion with IsoACh and atrial burst-pacing are known to induce calcium overload [25,26] as observed in AF and the combination of Ranolazine as ORM significantly suppressed AF inducibility by burst-pacing under IsoACh-perfusion.

### 3.2. Comparison of Both NCX-Inhibitors

In both groups, perfusion with ranolazine and both NCX-inhibitors altered the electrophysiological parameters. Rano-ORM led to an aPRR prolongation without reaching statistical differences while the number of AF episodes was significantly reduced. In contrast, Rano-SEA led to a statistically significant prolonged aPRR while there was only a trend towards a reduced number of AF episodes. This might be due to a comparatively low number of AF episodes after IsoACh-perfusion in the Rano-SEA group compared to the Rano-ORM group (26 episodes in the Rano-SEA group vs. 34 episodes in the Rano-SEA group). Furthermore, Rano-SEA shortened the aAPD compared to IsoACh-perfusion. A possible explanation for the differences between both NCX-inhibitors might be a different interaction with calcium channels, as Jost et al. [14] reported a significant L-type calcium channel inhibition of 10 µM SEA-0400 in contrast to a higher selectivity of ORM-10103 on the cardiac NCX-current.

### 3.3. Clinical Implications

With respect to the limitations mentioned above, non-invasive treatment options for AF are still limited. Therefore, many researchers are investigating new substances or substances approved for different indications. The observed electrophysiological effects of both combinations show a potential antiarrhythmic effect in different clinical settings. As reported previously, FDA-approved ranolazine can be used to terminate AF while it has lacking effect in the termination of persisting AF. As shown in our study, this combination might effectively suppress the occurrence of AF. Furthermore, a prolongation of aERP in the setting of an unaltered or even shortened aAPD seems to underline the potential antiarrhythmic effects in the persisting forms of AF. It has to be mentioned that the combination of ranolazine with NCX-inhibitors has not been tested previously and there are no data about the use of selective NCX-inhibitors in humans. In this context, a clinical application of ranolazine with NCX-inhibitors seems far away and further tolerability studies are needed.

### 3.4. Limitations

The data presented in this study were collected on isolated rabbit whole hearts. The transition to human hearts bears multiple limitations and detailed statements concerning the impact of ranolazine and NCX-inhibitors on specific ion currents cannot be made. Furthermore, the impact of each compound on the atrial electrophysiology cannot be dissected. However, the modes of action of ranolazine and NCX-inhibitors have been investigated before [3,4,13,14]. Of note, the induction of AF by perfusion with IsoACh does not represent the entire changes in atrial fibrillation and is likely to be associated with intracellular calcium overload [25], a situation in which NCX-inhibitors are expected to be very effective. Nonetheless, the isolated rabbit heart is frequently used to study drug interactions with the cardiac electrophysiology and is often employed in drug safety testing [27].

## 4. Methods

This study and the employed experimental protocol were approved by the local animal care committee (Landesamt für Natur, Umwelt und Verbraucherschutz Nordrhein-Westfalen, Germany, ID: 84-02.05.5016.004, date of approval: 20.04.2016) and conformed to the Guide for the Care and Use of Laboratory Animals published by the US National Institutes of Health (NIH Publication No. 852-3, revised 1996).

The preparation of the hearts was performed as already described [9]. To summarize, 18 female rabbits were anesthetized with sodium thiopental (200–300 mg i.v.) and exsanguinated. The animals were all females aging between 12 and 14 weeks and were supplied by Charles Rivers (Charles Rivers Laboratories, Wilmington, MA, USA). Hearts were harvested and mounted on a Langendorff apparatus (Hugo Sachs Electronic, Medical Research Instrumentation, March-Hugstetten, Germany) by cannulating the aorta. The spontaneously beating hearts were perfused at constant flow (52 mL/min) with warm (36.8–37.28 °C) Krebs–Henseleit solution (composition in mM: CaCl_2_ 1.80, KCl 4.70, KH_2_PO_4_ 1.18, MgSO_4_ 0.83, NaCl 118, NaHCO_3_ 24.88, Na-pyruvate 2.0, and D-glucose 5.55) at a pH around 7.4. The perfusate was oxygenated with 95% O_2_ and 5% CO_2_ (Linde Gas, Dublin, Ireland). The hearts were placed in a warming bath, filled with Krebs–Henseleit solution.

An electrocardiogram (ECG) was placed in the warming bath around the heart. Simultaneously, monophasic action potentials (MAP) recording and stimulation were accomplished by contact MAP catheters. A customized software allowed the analysis of the upstroke and the duration of the recorded action potentials. Five epicardial MAP catheters were placed on both atria with two recording MAPs on each atrium and one MAP for stimulation at the interseptal region.

### Experimental Protocol

**Figure 5:**
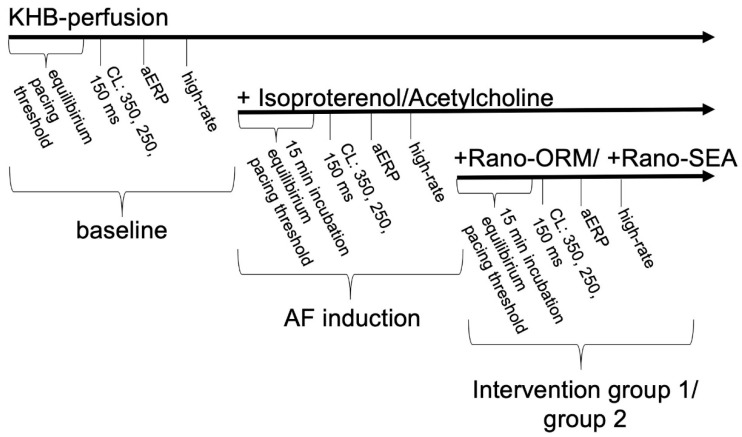
Schematic overview of the experimental protocol. KHB: Krebs-Henseleit-buffer; aERP: atrial effective refractory period; CL: cycle-length.

The experimental protocol is summarized in Figure 5. After placing the catheters, the diastolic pacing threshold was determined. The hearts were paced with a cycle-length (CL) of 350, 250, and 150 ms for 1 min to stabilize the action potential before recording 16 consecutive beats at each CL for action potential duration (APD) analysis. To determine the atrial effective refractory period (aERP) a single premature stimulus (S2) after an eight-beat train (S1–S1) was employed. The S1–S2 coupling interval was decremented in steps of 10 ms, starting with 130 ms. The shortest S1–S2 interval resulting in a propagated response was defined as aERP.

Thereafter, a standardized burst pacing protocol was performed to provoke AF. Burst pacing was conducted at 4, 8, and 12 times the diastolic threshold. For each amplitude, a train of ten 50 Hz bursts (1 s duration) at the basic CL of 250 ms with 10 s interval was delivered. Regarding the duration of AF, the episodes were defined as “non-sustained” (<1 s, minimum five beats) and “sustained” (≥1 s).

After the determination of the electrophysiological determinants at baseline, hearts were perfused with a combination of isoproterenol (1 µM) and acetylcholine (1 µM) to increase the occurrence of AF. The combination of both drugs has been previously tested on this [5] and other experimental models [28]. After 15 min of incubation, the aforementioned pacing protocol was employed again. Afterwards, the hearts were assigned to two groups. Group A (*n* = 9) was, additionally to IsoACh, perfused with 10 µM ranolazine and 1 µM ORM-10103 (group A: Rano-ORM) and the pacing protocol was employed for a third time after 15 min incubation. In group B (*n* = 9), the hearts were perfused with 10 µM ranolazine and 1 µM SEA0400 (group B: Rano-SEA) for 15 min before repassing the pacing protocol.

The observed data were entered into a computerized database (Microsoft Excel 16.35) and statistically evaluated using SPSS Software Version 25 (SPSS, Chicago, IL, USA). The atrial post repolarization refractoriness (aPRR) was calculated as aPRR = aERP−atrial action potential durations (aAPD_90_). Drug effects on aAPD_90_, aERP, aPRR, and the incidence of atrial arrhythmias were analysed employing the Wilcoxon test for paired groups. *p*-values < 0.05 were considered to be statistically significant. The sample size was calculated in accordance with previous studies of our group using the same experimental model.

## 5. Conclusions

To our knowledge, this is the first study investigating the effect of ranolazine combined with different selective NCX-inhibitors in an isolated whole-heart model of AF. Both combinations prolonged the atrial effective refractory period and reduced the incidence of AF episodes. Combining novel cellular targets may therefore lead to new potentially interesting options for antiarrhythmic AF therapy that remain to be tested in clinical studies.

## References

[1] Edvardsson N., Westlund A., Thimell M., Rise K., Todoran A., Aberg Kuren T., Kindblom J., Almgren O. (2010). Pharmacological rhythm and rate control treatment for atrial fibrillation: Patient and physician satisfaction. Patient.

[2] Voigt N., Heijman J., Dobrev D. (2015). New antiarrhythmic targets in atrial fibrillation. Future Cardiol..

[3] Gupta T., Khera S., Kolte D., Aronow W.S., Iwai S. (2015). Antiarrhythmic properties of ranolazine: A review of the current evidence. Int. J. Cardiol..

[4] Ratte A., Wiedmann F., Kraft M., Katus H.A., Schmidt C. (2019). Antiarrhythmic Properties of Ranolazine: Inhibition of Atrial Fibrillation Associated TASK-1 Potassium Channels. Front. Pharmacol..

[5] Frommeyer G., Schmidt M., Clauss C., Kaese S., Stypmann J., Pott C., Eckardt L., Milberg P. (2012). Further insights into the underlying electrophysiological mechanisms for reduction of atrial fibrillation by ranolazine in an experimental model of chronic heart failure. Eur. J. Heart Fail..

[6] Miles R.H., Passman R., Murdock D.K. (2011). Comparison of effectiveness and safety of ranolazine versus amiodarone for preventing atrial fibrillation after coronary artery bypass grafting. Am. J. Cardiol..

[7] Ramirez R.J., Takemoto Y., Martins R.P., Filgueiras-Rama D., Ennis S.R., Mironov S., Bhushal S., Deo M., Rajamani S., Berenfeld O. (2019). Mechanisms by Which Ranolazine Terminates Paroxysmal but Not Persistent Atrial Fibrillation. Circ. Arrhythm. Electrophysiol..

[8] Frommeyer G., Milberg P., Uphaus T., Kaiser D., Kaese S., Breithardt G., Eckardt L. (2013). Antiarrhythmic effect of ranolazine in combination with class III drugs in an experimental whole-heart model of atrial fibrillation. Cardiovasc. Ther..

[9] Carstensen H., Kjaer L., Haugaard M.M., Flethoj M., Hesselkilde E.Z., Kanters J.K., Pehrson S., Buhl R., Jespersen T. (2018). Antiarrhythmic Effects of Combining Dofetilide and Ranolazine in a Model of Acutely Induced Atrial Fibrillation in Horses. J. Cardiovasc. Pharmacol..

[10] Bogeholz N., Pauls P., Kaese S., Schulte J.S., Lemoine M.D., Dechering D.G., Frommeyer G., Goldhaber J.I., Seidl M.D., Kirchhefer U. (2016). Triggered activity in atrial myocytes is influenced by Na(+)/Ca(2+) exchanger activity in genetically altered mice. J. Mol. Cell Cardiol..

[11] Christ T., Kovacs P.P., Acsai K., Knaut M., Eschenhagen T., Jost N., Varro A., Wettwer E., Ravens U. (2016). Block of Na(+)/Ca(2+) exchanger by SEA0400 in human right atrial preparations from patients in sinus rhythm and in atrial fibrillation. Eur. J. Pharmacol..

[12] Lugenbiel P., Wenz F., Govorov K., Schweizer P.A., Katus H.A., Thomas D. (2015). Atrial fibrillation complicated by heart failure induces distinct remodeling of calcium cycling proteins. PLoS ONE.

[13] Bourgonje V.J., Vos M.A., Ozdemir S., Doisne N., Acsai K., Varro A., Sztojkov-Ivanov A., Zupko I., Rauch E., Kattner L. (2013). Combined Na(+)/Ca(2+) exchanger and L-type calcium channel block as a potential strategy to suppress arrhythmias and maintain ventricular function. Circ. Arrhythmia Electrophysiol..

[14] Jost N., Nagy N., Corici C., Kohajda Z., Horvath A., Acsai K., Biliczki P., Levijoki J., Pollesello P., Koskelainen T. (2013). ORM-10103, a novel specific inhibitor of the Na+/Ca2+ exchanger, decreases early and delayed afterdepolarizations in the canine heart. Br. J. Pharmacol..

[15] Frommeyer G., Mittelstedt A., Wolfes J., Ellermann C., Kochhauser S., Leitz P., Dechering D.G., Eckardt L. (2017). The anti-influenza drug oseltamivir reduces atrial fibrillation in an experimental whole-heart model. Naunyn Schmiedebergs Arch. Pharmacol..

[16] Ellermann C., Kohnke A., Dechering D.G., Kochhauser S., Reinke F., Fehr M., Eckardt L., Frommeyer G. (2018). Ranolazine Prevents Levosimendan-Induced Atrial Fibrillation. Pharmacology.

[17] Frommeyer G., Ellermann C., Dechering D.G., Kochhauser S., Bogeholz N., Guner F., Leitz P., Pott C., Eckardt L. (2016). Ranolazine and Vernakalant Prevent Ventricular Arrhythmias in an Experimental Whole-Heart Model of Short QT Syndrome. J. Cardiovasc. Electrophysiol..

[18] Gong M., Zhang Z., Fragakis N., Korantzopoulos P., Letsas K.P., Li G., Yan G.X., Liu T. (2017). Role of ranolazine in the prevention and treatment of atrial fibrillation: A meta-analysis of randomized clinical trials. Heart Rhythm..

[19] Sossalla S., Kallmeyer B., Wagner S., Mazur M., Maurer U., Toischer K., Schmitto J.D., Seipelt R., Schondube F.A., Hasenfuss G. (2010). Altered Na(+) currents in atrial fibrillation effects of ranolazine on arrhythmias and contractility in human atrial myocardium. J. Am. Coll. Cardiol..

[20] Burashnikov A. (2017). Late INa Inhibition as an Antiarrhythmic Strategy. J. Cardiovasc. Pharmacol..

[21] Frommeyer G., Rajamani S., Grundmann F., Stypmann J., Osada N., Breithardt G., Belardinelli L., Eckardt L., Milberg P. (2012). New insights into the beneficial electrophysiologic profile of ranolazine in heart failure: Prevention of ventricular fibrillation with increased postrepolarization refractoriness and without drug-induced proarrhythmia. J. Card Fail..

[22] Reiffel J.A., Camm A.J., Belardinelli L., Zeng D., Karwatowska-Prokopczuk E., Olmsted A., Zareba W., Rosero S., Kowey P., Investigators H. (2015). The HARMONY Trial: Combined Ranolazine and Dronedarone in the Management of Paroxysmal Atrial Fibrillation: Mechanistic and Therapeutic Synergism. Circ. Arrhythmia Electrophysiol..

[23] Hove-Madsen L., Llach A., Bayes-Genis A., Roura S., Rodriguez Font E., Aris A., Cinca J. (2004). Atrial fibrillation is associated with increased spontaneous calcium release from the sarcoplasmic reticulum in human atrial myocytes. Circulation.

[24] Voigt N., Li N., Wang Q., Wang W., Trafford A.W., Abu-Taha I., Sun Q., Wieland T., Ravens U., Nattel S. (2012). Enhanced sarcoplasmic reticulum Ca2+ leak and increased Na+-Ca2+ exchanger function underlie delayed afterdepolarizations in patients with chronic atrial fibrillation. Circulation.

[25] Wolkowicz P.E., Grenett H.E., Huang J., Wu H.C., Ku D.D., Urthaler F. (2007). A pharmacological model for calcium overload-induced tachycardia in isolated rat left atria. Eur. J. Pharmacol..

[26] Heijman J., Voigt N., Nattel S., Dobrev D. (2014). Cellular and molecular electrophysiology of atrial fibrillation initiation, maintenance, and progression. Circ. Res..

[27] Valentin J.P., Hoffmann P., De Clerck F., Hammond T.G., Hondeghem L. (2004). Review of the predictive value of the Langendorff heart model (Screenit system) in assessing the proarrhythmic potential of drugs. J. Pharmacol. Toxicol. Methods.

[28] Sicouri S., Gianetti B., Zygmunt A.C., Cordeiro J.M., Antzelevitch C. (2011). Antiarrhythmic effects of simvastatin in canine pulmonary vein sleeve preparations. J. Am. Coll. Cardiol..

